# Centering marginalized care: Home care cooperatives and system change

**DOI:** 10.1093/haschl/qxae184

**Published:** 2025-03-17

**Authors:** Geoffrey M Gusoff, Lina Stepick, Aquilina Soriano-Versoza, Katrina Kazda

**Affiliations:** Department of Family Medicine, David Geffen School of Medicine at UCLA, Los Angeles, CA 90095, USA; PHI, New York, NY 10016, USA; Pilipino Workers Center of Southern California, Los Angeles, CA 90026, USA; ICA Group, Northampton, MA 01060, USA

**Keywords:** home care, home care workers, system change, worker-ownership, political economy

## Abstract

Home care workers (HCWs), who provide paid in-home support for daily activities, are at the center of the care received by millions of Americans. However, HCWs are profoundly marginalized professionally and economically within our political-economic system, which devalues care work, public goods, and the labor of women, immigrants, and workers of color. This systemic marginalization has contributed to the impoverishment of millions of HCWs and massive workforce shortages, which prevent millions of Americans from accessing the consistent care they need. Home care cooperatives—businesses co-owned and controlled by HCWs—represent an alternative approach that places HCWs at the center. By providing greater compensation, training opportunities, and control over workplace decisions, home care cooperatives have achieved greater continuity of care and half the turnover rates compared with traditional agencies. They demonstrate what is possible when HCWs are centered at an organizational level and what could be achieved if HCWs were centered at a system level. This latter possibility requires the following: (1) reclaiming care work as a public good and investing in it accordingly; (2) structurally empowering HCWs within the care team and broader economy; and (3) new narratives about HCWs that recognize their skills, value, and centrality in providing quality care.

## Teaser

Home care workers, who provide paid in-home support with daily activities, provide critical support for millions of Americans, but they are structurally marginalized within our current political-economic system. This has resulted in a home care crisis marked by poverty wages for HCWs, massive workforce shortages, and HCW turnover rates up to 82% per year. In this commentary, we argue that home care cooperatives, which are owned and controlled by HCWs themselves, demonstrate what is possible at a system level when care work and caregivers are at the center. Through greater HCW compensation, training, and workplace control, these cooperatives have achieved higher continuity of care and half the turnover rates compared with traditional agencies. Replicating these results at scale requires a system that treats care work as a public good and invests in it accordingly, empowers HCWs within the care team, and promotes narratives about HCWs that recognize their skills, value, and centrality in providing care.

The 2.9 million HCWs in the United States provide paid in-home support with daily activities for millions of older adults and people with disabilities.^1^ They are one of the largest and fastest growing workforces, projected to add 781 000 jobs in the next decade.^2,3^ By many measures, HCWs are at the center of care, providing life-sustaining support and spending significantly more time with their clients than any other member of the healthcare team.^4^

However, HCWs are profoundly marginalized within the healthcare system and broader economy. Economically, their jobs are marked by irregular hours, scant benefits, and the lowest wages in healthcare.^5-9^ Professionally, they are systematically excluded from the care team despite their intimate knowledge of their clients.^4,10-13^ Culturally, they are persistently disrespected in a system that considers them unskilled and replaceable.^6,14,15^

##  

### The systemic roots of HCW marginalization

This systematic marginalization of HCWs is neither inevitable nor incidental. Rather, it is rooted in fundamental aspects of the U.S. political-economic system. First, the system's devaluation of care work as not “productive” and as subordinated feminine work contributes to chronic underinvestment in essential care sectors.^16^ Second, its devaluation of public goods—communal resources with broad benefits beyond the immediate participants—also contributes to chronic underinvestment in the care infrastructure required to support both economic productivity and personal dignity.^17^ Third, the system's reproduction of racial, gender, and nationality status hierarchies through occupational segregation devalues the labor of women, workers of color, and immigrants, who comprise the vast majority of the HCW workforce.^17,18^

### The consequences of HCW marginalization

The consequences of this systemic HCW marginalization are dire for HCWs, care recipients, and the broader community. Due to poor HCW wages and working conditions, HCWs have among the highest rates of workplace injuries and nearly 40% of HCWs have household incomes <200% of the federal poverty line.^19,20^

This has contributed to HCW turnover rates of up to 82% per year and massive workforce shortages.^21^ At a time when the nation's demand for HCW services is at an all-time high, census data suggest the per capita supply of HCWs is at a historic low.^22^

This shortage puts immense strain on care recipients, their family members, the healthcare system, and the broader economy. Family caregivers, predominantly women, are often forced to leave their jobs or reduce hours. High turnover also undermines the consistency required for quality care.^23,24^ A recent analysis by Jabola-Carolus et al.^25^ estimates HCW turnover costs to be approximately 16% of median HCW earnings, suggesting overall HCW turnover costs of $6.6-8.4 billion per year, not counting the additional burden of preventable hospitalizations and nursing home stays for those unable to access needed home care.^21^

In summary, our current care crisis is a systemic crisis, rooted in racism, sexism, and other structural pathologies in our political-economic system, which devalues care work and those who perform it. Overcoming the crisis requires system alternatives that center care work and care workers. Home care cooperatives, businesses co-owned and controlled by HCWs themselves, embody that type of alternative.

## Home care cooperatives: placing HCWs at the center of care

The first US home care cooperative, Cooperative Home Care Associates (CHCA), was founded in the Bronx in 1985 with a group of 12 HCWs. Since then, it has grown to employ >1400 HCWs. Cooperative Home Care Associates's mission is to provide quality care through quality jobs, improving HCWs' dismal work conditions by placing them at the center of the organization.^26^

In the cooperative model, HCWs are the agency's primary owners and the ultimate beneficiaries of any profits.^27^ Home care workers make up the majority of the cooperative's governing board, participating in major operational and financial decisions such as determining wage rates, worker benefits, and safety protocols. Home care workers also participate in these decisions through committees and member-wide voting.^28^ The cooperative includes non-HCW members (eg, supervisors, managers), but these staff are ultimately accountable to the HCW-majority board and many are former HCWs themselves.

Cooperative Home Care Associates has become a national model for HCW job quality and care quality, offering a premier 4-week hands-on training program, comprehensive benefits, and a guaranteed hours program.^29^

The home care cooperative model has also spread across the country, with 22 home care cooperatives ranging from rural to urban settings, small to large agency size, and Medicaid-funded to private pay.^30^ What they all share is a model that centers HCWs as co-owners and key decision-makers.

### Impacts of the home care cooperative model

Across various settings, the home care cooperative model has demonstrated impressive results in improving HCW job quality and care quality. Compared with traditional agencies, where HCWs typically have little decision-making authority and no ownership stake, home care cooperatives have higher overall compensation in the form of higher wages ($2.01 more per hour on average), more hours, better benefits, and/or profit-sharing.^31,32^ Home care workers at cooperatives also report higher levels of support, respect, and voice in workplace decisions, critical factors for improving job satisfaction and retention.^28,33,34^ As a result, cooperatives have achieved HCW turnover rates that are half the industry average.^35^

Regarding care quality, cooperatives' lower turnover rates facilitate more consistent care for clients, which is particularly important in long-term care.^23^ Cooperatives also tend to make greater investments in hands-on and extensive HCW training, which has been associated with improved care.^36^ Finally, cooperatives emphasize frontline worker input into care planning, which is associated with greater patient safety.^24,37^ These factors help explain why clients stay longer with cooperatives than with traditional agencies.^38^

### How home care cooperatives center marginalized care

Home care cooperatives have achieved these improvements in job quality and care quality by centering HCWs in ways they have traditionally been marginalized. Economically, co-ownership makes HCWs the ultimate beneficiaries of profits, so surpluses go primarily toward HCW wages, benefits, and training instead of outside investors or outsized managerial salaries.

Professionally, cooperatives prioritize HCW input in care planning and other workplace decisions. This occurs through regular communication between HCWs and supervisors or specialized roles like care navigators facilitating HCW input to office-based staff. Through participation in the board or committees, HCWs also help create care-related policies. In these ways, HCWs at cooperatives are more integrated into the broader care team.

Culturally, HCWs report a much stronger culture of respect at cooperatives than at traditional agencies. The cooperative structure itself, in which HCWs have significant input into workplace policies and oversight of managerial staff, is an important contributor to this culture. This organizational structure of HCW leadership also disrupts traditional status hierarchies, centering the leadership of women, people of color, and immigrants. Cooperatives' collaborative approaches to supervision and hiring of former HCWs into office roles also increase mutual understanding and respect.^39,40^

By centering HCWs economically, professionally, and culturally, home care cooperatives have pushed back against the systemic forces that marginalize this workforce.

## Applying the lessons of home care cooperatives: centering care through system change

The experience of home care cooperatives provides key lessons for the home care sector. First, investing in HCW compensation and training can have substantial returns in worker retention, job quality, and care quality. Second, HCWs are capable of and desire more central roles in the care team, providing critical input that improves both their jobs and the care they provide. Third, cooperatives embody a powerful alternative narrative that can guide system design: HCWs as skilled collaborators with unique contributions that the system ignores at its peril.

Applying these lessons to transform our current system of care requires not only scaling up the cooperative model but also structural changes across the entire sector. Despite significant recent growth in home care cooperatives, they currently employ only 0.1% of all HCWs, with regulatory and financial barriers slowing cooperative development.^41^ These include a reluctance among traditional banks to lend to cooperatives, limited equity contributions and concerns around debt-financing among low-income HCWs, Medicaid billing requirements and payment delays requiring significant administrative resources and working capital (particularly challenging for smaller cooperatives), and a lack of cooperative-specific training and technical assistance resources.

Many of these barriers are currently being remedied through the development of cooperative-specific loan funds and secondary cooperatives that centralize administrative functions across cooperatives and provide cooperative-specific training and technical assistance, contributing to exponential growth among developing cooperatives. However, truly centering HCWs within cooperatives and across the home care industry requires system changes that build on cooperatives' successes.

### Centering HCWs economically

While home care cooperatives have significantly improved HCW job quality and care quality by focusing their limited revenues on HCW compensation and supports, low reimbursement rates from public payers and the unaffordability of private pay home care for most households make it impossible for even cooperatives to provide HCWs living wages with full benefits and high-quality training. Greater public system-wide investments are needed to compensate HCWs commensurate with the value they provide and to sustain the high-quality HCW workforce needed to meet demand.

This requires investing in home care as a public good rather than as a private commodity. A public good approach, such as treating care work as “infrastructure”, better captures the social value of care work for recipients, unpaid caregivers, the healthcare system, and the economy, recognizing its direct contributions to recipients and the way it facilitates all other forms of work.

Increasing Medicaid and Medicare HCW reimbursement rates to enable livable wages and a sustainable workforce is crucial for repairing our crumbling care infrastructure. In addition, public long-term care insurance programs—key sources of long-term care financing in other OECD countries and recently introduced in Washington state—should be developed and expanded to ensure affordable care coverage across the income spectrum. Expanding minimum wage laws covering HCWs, wage pass-through laws requiring a specified portion of reimbursement goes to HCW wages, and worker empowerment efforts (ie, collective bargaining, board participation) can help ensure these increased investments actually reach HCWs as they do at cooperatives.^42-44^ Finally, public agencies should increase investments in standardized, high-quality HCW trainings to ensure a well-trained workforce.

### Centering HCWs professionally

Home care workers must also be empowered within the broader health care team, participating in organizational and care decisions. Cooperatives represent one effective approach for institutionalizing this participation, as do co-determination models in which HCWs serve on the agency's board and unionization through which HCWs participate on labor-management committees and engage in policy advocacy, among other union benefits.

New value-based payment models, such as pay for performance and shared savings programs that reward HCWs for high-quality care and reducing unnecessary costs, can encourage better coordination across the care team to meet quality and value goals.^36,45^ By rewarding higher quality, these approaches can also promote the growth of home care cooperatives and other high-performing home care models.

These structural approaches to HCW empowerment should also be complemented with HCW-centering care team practices. Collaborative approaches to HCW supervision, developed by cooperatives and linked to improved teamwork and job satisfaction, should be implemented broadly.^46^ The development of advanced caregiver roles, associated with improved HCW and client outcomes, should also be adopted.^47,48^ Finally, HCW-based interventions, such as those being studied around heart failure and physical activity, should be expanded to more fully utilize HCWs' skills.^49,50^

### Centering HCWs culturally

Finally, it's important that these HCW-centering practices are accompanied by HCW-centering narratives. Care work as “infrastructure” helpfully highlights the essential nature of HCWs but must also emphasize HCWs do not merely facilitate others' labor force participation but also perform critical, skilled work as care professionals. These and other framings must emphasize the centrality of HCWs' contributions to individuals, communities, and the economy.

## Conclusion: building a caring political economy

A system that truly takes care of its people must also take care of its caregivers. The fact that our system does just the opposite is neither an accident nor an inevitability—it is rooted in a fundamental political-economic orientation that needs to be transformed. Home care cooperatives embody an alternative approach to care and point to key components of needed system change: investing in care work as a public good, empowering HCWs within the care team, and promoting HCW-centric narratives. Following their lead, we can begin to build a system where better care is not only possible but deeply embedded.

## Supplementary Material

qxae184_Supplementary_Data
